# Validation of microRNA expression profile in Oral Lichenoid Disease through cytological samples

**DOI:** 10.4317/medoral.23020

**Published:** 2019-08-19

**Authors:** Amaia Setién-Olarra, Xabier Marichalar-Mendia, Juan-Alonso Fernández-Pacheco, Marcos Fernández-Barriales-López, María-Luisa Gainza-Cirauqui, José-Manuel Aguirre-Urizar

**Affiliations:** 1 Oral Medicine and Pathology, Department of Stomatology II, UFI 11/25, University of the Basque Country (UPV/EHU). Leioa, Spain; 2 Servicio de Cirugía Maxilofacial. Hospital Universitario Araba (Osakidetza. OSI Araba). Vitoria-Gazteiz, Spain; 3 Department of Dental Surgery, Faculty of Dental Surgery, University of Malta. Msida, Malta

## Abstract

**Background:**

To validate oral exfoliative cytology in the analysis of the microRNA expression profile in Oral Lichenoid Disease (OLD).

**Material and Methods:**

The expression of 13 microRNAs identified and presented by our group in a previous study was analyzed in 26 cases, 16 diagnosed as OLD and 10 controls with no oral mucosal pathology. Cytological samples from the oral mucosa obtained using an Orcellex toothbrush were analyzed using RT-qPCR and TaqMan microRNA assays.

**Results:**

The aberrant expression was validated for 2 microRNAs (miR-146a-5p and miR-7-1-3p) of those previously recognized in the biopsy study.

**Conclusions:**

This is the first time that oral exfoliative cytology is validated in a study of the alterations of the expression of microRNAs in OLD. The alteration of miR-146a and miR-7 compared to controls was validated. These microRNAs are associated with both inflammatory and carcinogenic phenomena that are involved in the etiopathogenesis of this potentially malignant oral disorder.

** Key words:**microRNAs, lichen planus, epigenetics, cytological techniques.

## Introduction

Oral lichenoid disease (OLD) is a chronic inflammatory disorder with an immunological base that is present in nearly 2% of the population, mainly affecting adult women (1,2). This disease is considered a potentially malignant oral disorder with a variable percentage of malignant transformation (0.4-6.5%) (3). To date, data that can predict the risk of malignant transformation of this disease and that help establish effective preventive measures to avoid such transformation remain unreliable (2,4).

In recent years, the interest in the use of oral exfoliative cytology has grown significantly. This minimally invasive method helps us to obtain epithelial cells for the diagnosis of oral precancerous processes and for their molecular analysis (5-7).

MicroRNAs are non-coding RNA molecules that act in post-transcriptional regulation of gene expression and are partially complementarily bonded to the 3´region of the messenger RNA causing transcriptional repression or direct degradation of the mRNA (8,9). It is considered that the nearly 2000 microRNAs described can regulate the expression of 60% of the human genes and that they can also regulate biological processes such as growth, differentiation and cellular death (9). Evidence shows that microRNAs play an important role in cancer, possibly acting as oncogenes or tumor suppressor genes (10). Studies also outline changes in the expression profile of these microRNAs during the malignant transformation of premalignant lesions into oral cancer (11,12). These molecules are very stable and can be easily detected in various media such as paraffin-embedded tissue samples, biological fluids and cytological samples, widening their potential for analysis (13,14).

Until now, few studies (15-17) have analyzed the expression profile of microRNAs in OLD, yet none of them using oral cytological samples. Obtaining RNA with a non-surgical approach may be ideal in detecting this potentially malignant oral disorder and its potential of malignant transformation.

In 2016, our group analyzed the expression of 768 mature microRNAs in the highest number of OLD samples studied so far (17). Of the 20 deregulated microRNAs, the 13 with a higher degree of deregulation were further validated in an independent set of samples.

With this background, we designed a study with the aim of assessing the viability of exfoliative cytology samples for the analysis of microRNA expression profile in OLD and its use in its diagnosis and its prognosis.

## Material and Methods

-Patients and samples

Oral cytology samples from 26 individuals were obtained from Oral Medicine Unit of the Dental Clinical Service (University of the Basque Country/EHU) and the Maxillofacial Surgery Service (Hospital Universitario Araba-Osakidetza). The OLD group comprised 16 patients (mean age, 63.7 years; range 45–78 years; female-to-male ratio, 3:1) with clinical and histopathological diagnosis of OLD, according to the criteria previously described (1). The study included a control group that comprised 10 healthy patients without oral mucosa disorders (mean age, 57.5 years; range, 51–71 years; female-to-male ratio, 4:1). Demographic characteristics are shown in  Table 1. None of the patients were smokers, consumed alcohol or were in any drug treatment. A cytological brush (Orcellex Brush, Rovers Medical Devices, The Netherlands) was vigorously swabbed over the lesions in OLD and over the buccal and lingual mucosa in control group. The brush head was introduced into a sterile tube with RNA lysis buffer (Zymo Research, USA) and stored at -20 ° C.

**Table 1:**
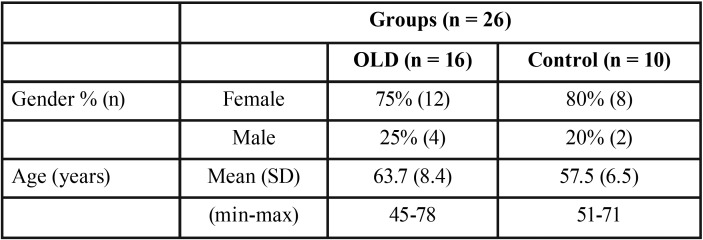
Demographic characteristics of the patients included in the study.

This study was carried out in accordance with the principles of the Declaration of Helsinki on Ethical Principles for Medical Research Involving Human Subjects and was approved by the Ethics Committee for Research of the University of the Basque Country/ EHU (CEISH/271/2014). In this study the methodology followed was similar as previously described by Setién-Olarra *et al.* (17).

RNA isolation and microRNA quantitative RT-PCR analysis

Total RNA including microRNA obtained by brush cytology was extracted using the Quick - RNA Miniprep kit (Zymo Research, USA). The purity and concentration of RNA were determined by OD260/280/230 readings using a NanoDrop ND-1000 spectrophotometer (Thermo Fisher Scientific, Eugene, OR, USA). RNA integrity was determined by fluorometric quantitation using the Qubit 3 Fluorometer (Life Technologies, Foster City, CA, USA).

Previously identified microRNAs were evaluated in an independent validation set of 26 oral cytology samples (16 OLD and 10 control) by RT-qPCR using TaqMan microRNA assays (Applied Biosystems). As reported (17), miR-30b, miR-26a, and miR-26b were proposed as candidate normalizers for the validation step. Each microRNA was spotted in triplicate, and no-template RT controls were carried along in each experiment. Ct values of the target microRNAs were normalized in relation to that of the three normalize microRNAs, and microRNA expression levels [relative quantity (RQ)] were calculated using the comparative Ct method: CtSample–CtMeannormalizers.

-Data analysis

Pairwise comparisons of quantitative data obtained by RT-qPCR experiments were made by the Mann–Whitney U test. All tests were two-sided, and microRNAs showing two-fold difference changes [fold change (FC) >2] between OLD vs normal and adjusted *P*-value <0.05 were considered statistically significant. A binary logistic regression analysis was performed in those cases were there was statistical significance. The statistical analyses in the validation phase were performed using SPSS v18.

## Results

-Validation of differently expressed microRNAs

Our previous study (17) using oral biopsy samples in patients diagnosed with OLD showed an altered expression profile in 13 microRNAs (microRNA-150-5p, microRNA-142-5p, microRNA-146a-5p, microRNA-223-5p, microRNA-7-1-3p, microRNA-339-3p, microRNA-342-3p, microRNA-146b-5p, microRNA-140-3p, microRNA-1247-5p, microRNA-152-3p, microRNA-625-5p, microRNA-629-3p). These 13 altered microRNAs were analyzed in the present study.  Table 2 shows the comparative results of these 13 microRNAs.

**Table 2:**
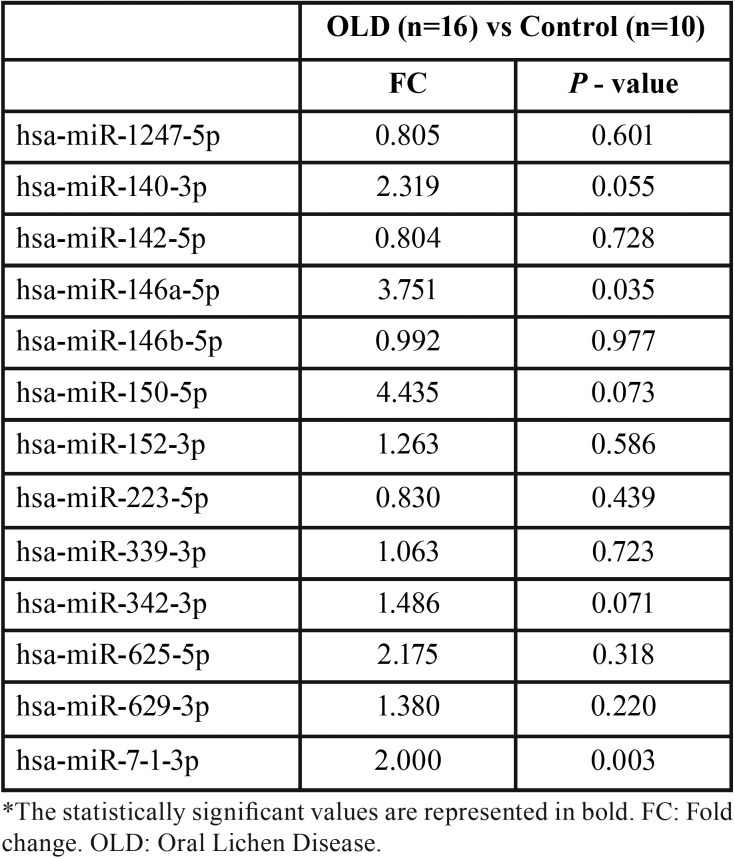
Biological validation of the 13 microRNAs selected.

We compared the results of the aberrant expression in cytological samples with those obtained in our previous study on biopsy samples (17), and we were able to validate significant differences in 2 microRNAs and showing a significant overexpression (*p*<0.05) in the OLD group compared to the control group (miR-7-1-3p, miR-146a-5p) ( Table 2). Specifically, OLD samples show a 3.7 times greater expression of miR-146a-5p than control samples (*p*=0.035). On the other hand, overexpression of miR-7-1-3p was 2-times greater in OLD when compared to the control group (*p*=0.003).

 Table 3 shows the results of the binary logistic regression analysis for the differently expressed microRNAs. Overexpression of miR-7-1-3p increases the risk of suffering OLD.

**Table 3:**
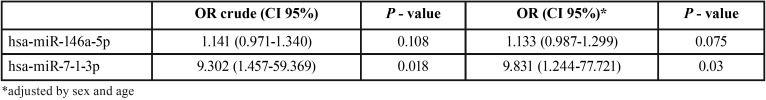
Binary logistic regression results for statistically significant microRNAs.

## Discussion

OLD is a potentially malignant oral disorder that has, in most cases, an unknown and/or uncertain etiology and a malignant potential that varies widely (3,18). In 2009, Cervigne *et al.* (11) suggested that microRNAs could be used to predict malignant transformation of premalignant oral lesions. Following this line, our group (17) analyzed the expression pattern of 768 microRNAs in a significant number of patients, validating the profile of 13 microRNAs with a different expression profile in the disease. Considering these results, we consider using oral cytology to diagnose and monitor these alterations with as a less invasive method and consequently, we aim to validate the results in oral cytological samples of a group of patients diagnosed with OLD.

In this study, we could only validate the alteration in the expression of 2 microRNAs (miR-146a-5p and miR-7-1-3p) when comparing the OLD group with the control group. We were unable to validate the remaining altered microRNAs from the previous study in this set of samples, which indicates the existence of differences in the analysis of biopsy and cytology samples from these lesions. We believe that this is mainly due to the difference in the composition of the tissue and cells in each type of sample. Exfoliative cytology of the oral mucosa includes mainly superficial cells of the scraped epithelial cells from the mucosa (mature keratinocytes) (5,19); while biopsy samples comprise the totality of the mucosa, including all the layers of epithelial cells, as well as connective tissue cells from the lamina propria. This situation was raised also by He *et al.* (7) in a study on tongue cancer samples in which the qualitative and quantitative differences in microRNA expression between the biopsy samples and cytology samples were observed.

MicroRNA-146a was validated extensively in our study and is the only microRNA that has been described in oral cancer and potentially malignant oral disorders in both complete genome and candidate gene studies (17,20,21). Its implication in inflammatory and immunological processes, as well as in various neoplasms including oral cancer, has been demonstrated (21-25). Recently, Zhang *et al.* (25) demonstrated that the allelic variants of miR-146a rs2910164 in a Chinese population may be associated with a higher risk of presenting head and neck squamous cell carcinoma. Based on these findings, we consider that microRNA-146a may be an important element in oral carcinogenesis by mediating the regulation of the proinflammatory pathways with a carcinogenic potential (26). Therefore, the early detection using a simple and non-invasive technique such as oral exfoliative cytology may contribute with valuable information in patients diagnosed with OLD in which we should apply careful monitoring.

Regarding microRNA-7, its deregulation has been recognized in different malignant neoplasms, among which the oral squamous cell carcinoma is included (27-29). In 2010, Jiang *et al.* (28) observed that microRNA-7 could cause the subexpression of the receptor of the IGF1R growth factor in cells of tongue squamous cell carcinoma and could mitigate phosphorylation or activation of the AKT kinase protein, a protein associated with inflammatory processes mediated by cytokines (30), causing a reduction in cell proliferation and an enhancement of apoptosis (28). Furthermore, our results show that overexpression of microRNA-7 increases the risk of presenting OLD. All this indicates that microRNA-7 may have an oncogenic regulating role and a role in regulating certain inflammatory processes, among which the chronic inflammatory process of OLD is included.

These initially promising results show certain limitations due to the retrospective aspect and the size of the sample. In future studies, we suggest that a bigger sample is used in order to obtain stronger statistical results. It is possible that this will provide us with more answers in other validated microRNAs, such as hsa-miR-150-5p, el hsa-miR-342-3p or hsa-miR-140-3p that showed p-values close to the 0.05 threshold.

In conclusion, our study is the first to analyze the viability of the oral exfoliative cytology for the study of the alterations of the expression of microRNAs in OLD. The alteration of miR-146a and miR-7 compared to controls was validated. The aberrant expression of these microRNAs may be used in the diagnosis of OLD and in the individual prognostic assessment.

Acknowledgments: This research was supported by a grant from Basque Goverment Health Department (2013111005). The authors are also grateful to the Biobanco Vasco de la Fundación Vasca de Innovación e Investigación (BIOEF) for kindly providing the samples.
